# Breaking the culture habit: Complementing culture-based veterinary diagnostics with metagenomic data -A case study of feline and canine skin infections

**DOI:** 10.1186/s12917-026-05476-x

**Published:** 2026-05-09

**Authors:** Andrea Ottesen, Brandon Kocurek, Mark K. Mammel, Sanchez Jn Charles, Jaclyn Dietrich, Sarah Pauley, Stephen D. Cole, Shelley Rankin, Olgica Ceric

**Affiliations:** 1 Center for Veterinary Medicine, Food and Drug Administration, Laurel, MD United States of America; 2 Human Foods Program, Food and Drug Administration, Laurel, MD United States of America; 3 University of Pennsylvania School of Veterinary Medicine, Philadelphia, PA United States of America; 4 Center for Veterinary Medicine, National Antimicrobial Resistance Monitoring System (NARMS), Food and Drug Administration, Laurel, MD United States of America; 5 Veterinary Laboratory Investigation and Response Network (Vet-LIRN), Center for Veterinary Medicine, Food and Drug Administration, Laurel, MD United States of America

**Keywords:** Veterinary diagnostics, Metagenomic data, Canine, Feline, Skin infections, Methicillin resistance, Antimicrobial resistance

## Abstract

**Background:**

Skin infections have been described as the primary cause for veterinary small animal practice visits, frequently requiring topical and systemic antibiotics. These infections often represent secondary complications of underlying pathologies, that can lead to recurrent infections and multiple antibiotic exposures. This creates selection pressure toward antibiotic resistance at the intersection of skin, bloodstream, and shared human-animal environments. This case study integrates Veterinary Diagnostic Laboratory (VDL) aerobic culture results with metagenomic (MGX) data to evaluate the combined utility of these approaches in advancing One Health veterinary diagnostics. Simultaneous reporting of culture-recovered pathogens alongside infection microbiomes and resistomes could strengthen pathogen epidemiology, illuminate polymicrobial etiologies, and inform antimicrobial stewardship.

**Results:**

One feline and eight canine skin swabs were analyzed with aerobic culture and traditional antimicrobial susceptibility testing (AST) and compared with MGX profiles. VDL aerobic culture and AST identified *Staphylococcus aureus*,* S. pseudintermedius*, *S. schleiferi*, methicillin resistant (MR) *S. schleiferi* (MRSS), MR *S. pseudintermedius* (MRSP) and *Pseudomonas aeruginosa*. MGX data detected the identical bacterial pathogens and identified methicillin resistance genes (*mecA*,* mecI*,* mecR1*) in samples where AST had confirmed MRSP and MRSS. MGX data also detected *mec* genes in samples without culture confirmed MR phenotypes as well as describing multi-domain microbiota (bacteria, fungi, protists, viruses, phages), antimicrobial resistance genes (ARGs), plasmids, and metabolic features associated with the skin infection samples.

**Conclusions:**

MGX data detected the identical VDL recovered pathogens and genes that confer the AMR phenotypes recovered by VDL AST. MGX data also detected additional uncultured pathogens, ARGs, multi-domain microbiota, mobile AMR elements, and metabolic features. Future applications for these methods used simultaneously could support monitoring programs, advance pathogen epidemiology, inform treatment strategy, advance judicious antimicrobial administration, and provide data for machine learning (ML) models to improve precision veterinary diagnosis and treatment.

**Supplementary Information:**

The online version contains supplementary material available at 10.1186/s12917-026-05476-x.

* The views expressed in this manuscript are those of the authors and do not necessarily reflect the official policy of the Department of Health and Human Services, the U.S. Food and Drug Administration, or the U.S. Government. Reference to any commercial materials, equipment, or process does not in any way constitute approval, endorsement, or recommendation by the Food and Drug Administration.

## Introduction

Companion animals’ skin infections have been described as the primary reason for presentation in small animal practices and result in frequent antibiotic administration [1]. Skin infections are often secondary complications of reduced immunity associated with underlying pathologies and/or dysbiosis, so precise diagnoses can be elusive. It is not uncommon for skin infections to recur, leading to subsequent *re-exposure* to antimicrobials. This scenario results in AMR selection pressures at the intersection of the skin (largest mammalian organ), the bloodstream, and shared human and animal environments [1]. Dogs and cats are often treated with the same antibiotics used in human medicine such as fluoroquinolones, penicillins, and cephalosporins [2] which, considered with the proximity shared by humans and companion animals, underscores the importance of AMR stewardship at this One Health nexus [3]. To reduce selection pressures for Difficult to Treat Resistance (DTR) pathogens, diagnostics which identify the full consortia of pathogens present in an infection with the total resistome, in a single step, will be crucial to the advancement of public health and antimicrobial stewardship [4].

Metagenomic data have been used to identify pathogens in human and animal diagnostics for etiologies of unknown origin for more than two decades [5–10]. Though work in the veterinary purview is more emergent, researchers have been advancing metagenomic-based veterinary diagnostics for detection of porcine viruses [11, 12], bovine respiratory disease [13], peri weaning failure-to-thrive syndrome (pigs) [14], shaking mink syndrome [8], meningoencephalomyelitis of unknown origin (dogs) [15], and cat scratch disease [16], to list a few. Viral diagnostics were some of the first implementations of MGX-based veterinary diagnostics in response to the challenges associated with culture and recovery of viruses [8, 17–20]. All assays are most powerful when optimized for a specific objective which is especially significant in the veterinary purview where multiple host species, multiple microbiomes, resistomes, and pathologies, provide considerable challenges for any single protocol.

Metagenomic data describe the total ecology of any environment, however there can be significant limitations when the aim is to detect low abundant organisms such as human or animal pathogens. Detection challenges vary in difficulty and resource intensity depending on complexity and diversity of sample environments, native abundance of pathogens, and precision of reference and analytic tools. To navigate these challenges for bacterial pathogens, shotgun sequencing of microbiological recovery enrichments at strategic temporal intervals during the enrichment processes has been used to increase pathogen DNA available for bioinformatic analysis. These approaches, referred to as quasi-metagenomics [21–23], have reduced foodborne illness outbreak response time [22], advanced understanding of serovar diversity associated with foodborne illness outbreaks [24], identified co-occurring pathogen antagonists and enrichment biases [25], and contributed to improved efficiency of state of the art pathogen recovery protocols. Here, we use metagenomic and quasi-metagenomic data alongside results from culture based VDL diagnostics of feline and canine skin infections to demonstrate how integration of these data types may synergistically advance pathogen diagnostics, epidemiology, One Health zoonotic risk assessment, and judicious antimicrobial treatment.

## Results

### Veterinary diagnostic laboratory (VDL) culture results

VDL aerobic culture results from swabs of skin infections of one cat and eight dogs recovered *Staphylococcus schleiferi*,* Staphylococcus aureus*, methicillin resistant *Staphylococcus pseudintermedius* (MRSP), methicillin resistant *Staphylococcus schleiferi* (MRSS) and *Pseudomonas aeruginosa.* VDL recovered pathogens are shown in Table 1 and complete antibiotic sensitivity testing (AST) profiles are available in Supplementary Materials (S1:_Full AST).

**Table 1:** VDL culture and AST results for skin infections from 1 cat and 8 dogs

ID #	Organism Recovered	Resistance to Antibiotic, (I) Intermediate Resistance
FS1	*Staphylococcus aureus*	Cefovecin (I), Cefpodoxime (I), Enrofloxacin
CS1	*Pseudomonas aeruginosa*	Cefovecin, Ceftiofur, Ciprofloxacin (I), Enrofloxacin (I),
CS1	*Staphylococcus schleiferi*	Enrofloxacin
CS2	*Pseudomonas aeruginosa*	Cefovecin, Ceftiofur, Ciprofloxacin (I), Enrofloxacin (I),
CS3	*Pseudomonas aeruginosa*	Cefovecin, Ceftiofur, Ciprofloxacin (I), Enrofloxacin (I),
CS4	*MR Staphylococcus schleiferi*	Cefovecin, Clindamycin, Cefpodoxime, Doxycycline, Erythromycin, Enrofloxacin (I), Marbofloxacin (I), Oxacillin, Benzylpenicillin
CS5	*MR Staphylococcus pseudintermedius*	Doxycycline, Enrofloxacin, Gentamicin (I), Minocycline, Marbofloxacin, Oxacillin, Benzylpenicillin, Pradofloxacin, Trimethoprim/Sulfamethoxazole
CS6	*Staphylococcus pseudintermedius*	Doxycycline, Minocycline, Benzylpenicillin
CS7	*Staphylococcus pseudintermedius*	Benzylpenicillin
CS8	*MR Staphylococcus schleiferi*	Cefovecin, Cefpodoxime (I), Enrofloxacin, Marbofloxacin, Oxacillin, Benzylpenicillin, Pradofloxacin (I)

### Metagenomic profiling

**Fig. 1:**
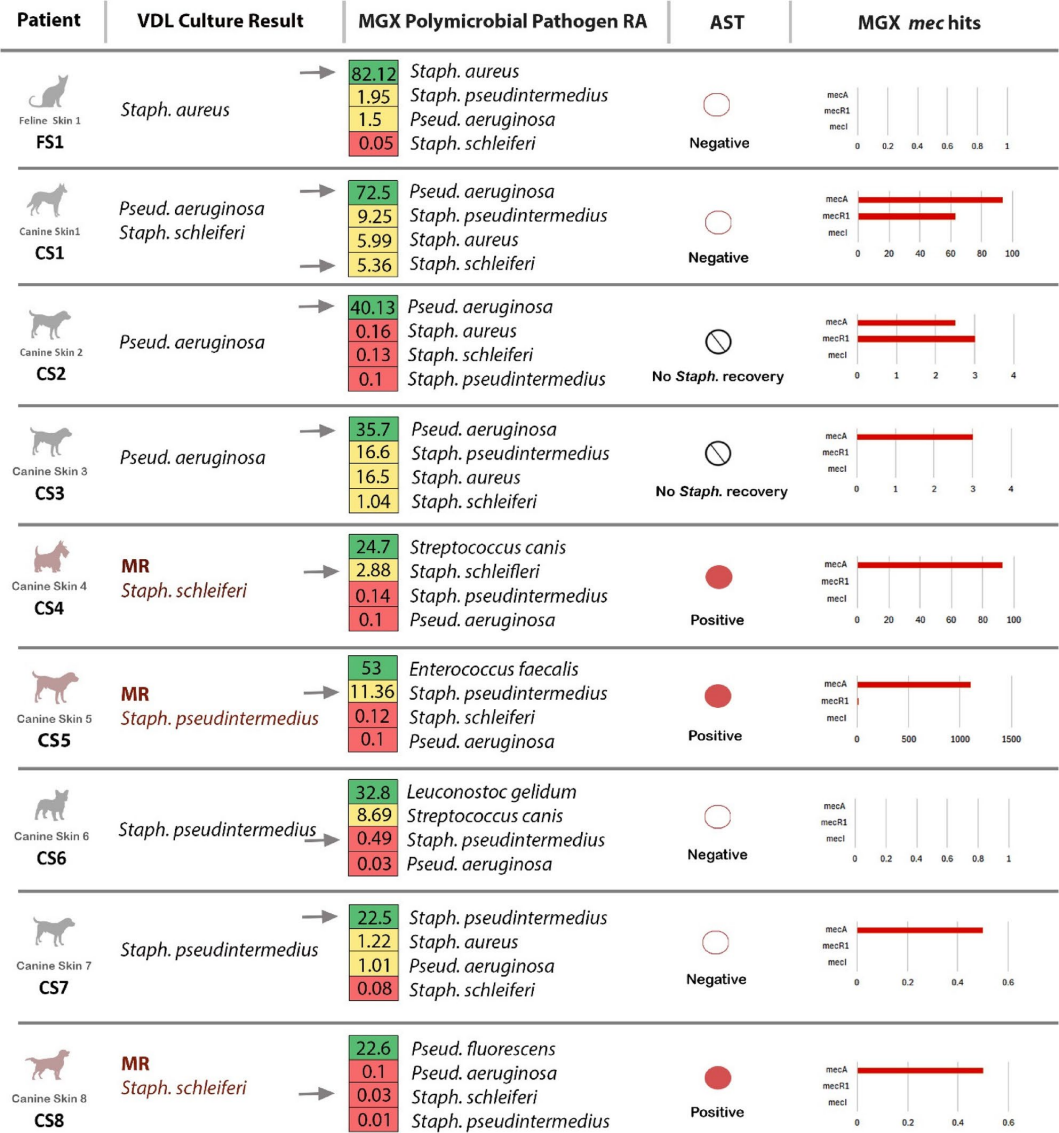
Summary of Culture and Metagenomic Results Figure 1 shows the number of each feline and canine patient along the left side of the panel. Across the figure for each sample are: VDL aerobic pathogen recovery results, polymicrobial relative abundance (RA) of important pathogens observed in MGX data. Green cells highlight the RA of the most abundant pathogen, followed by yellow, representing reduced abundance of other key pathogens and red cells highlight pathogens observed at RA < 1%. The last two columns show concordance of VDL AST isolate phenotypes and abundance of MGX detected methicillin resistance genes *mec*A, *mec*R1, and *mec*I normalized and averaged for each sample

**Fig. 2:**
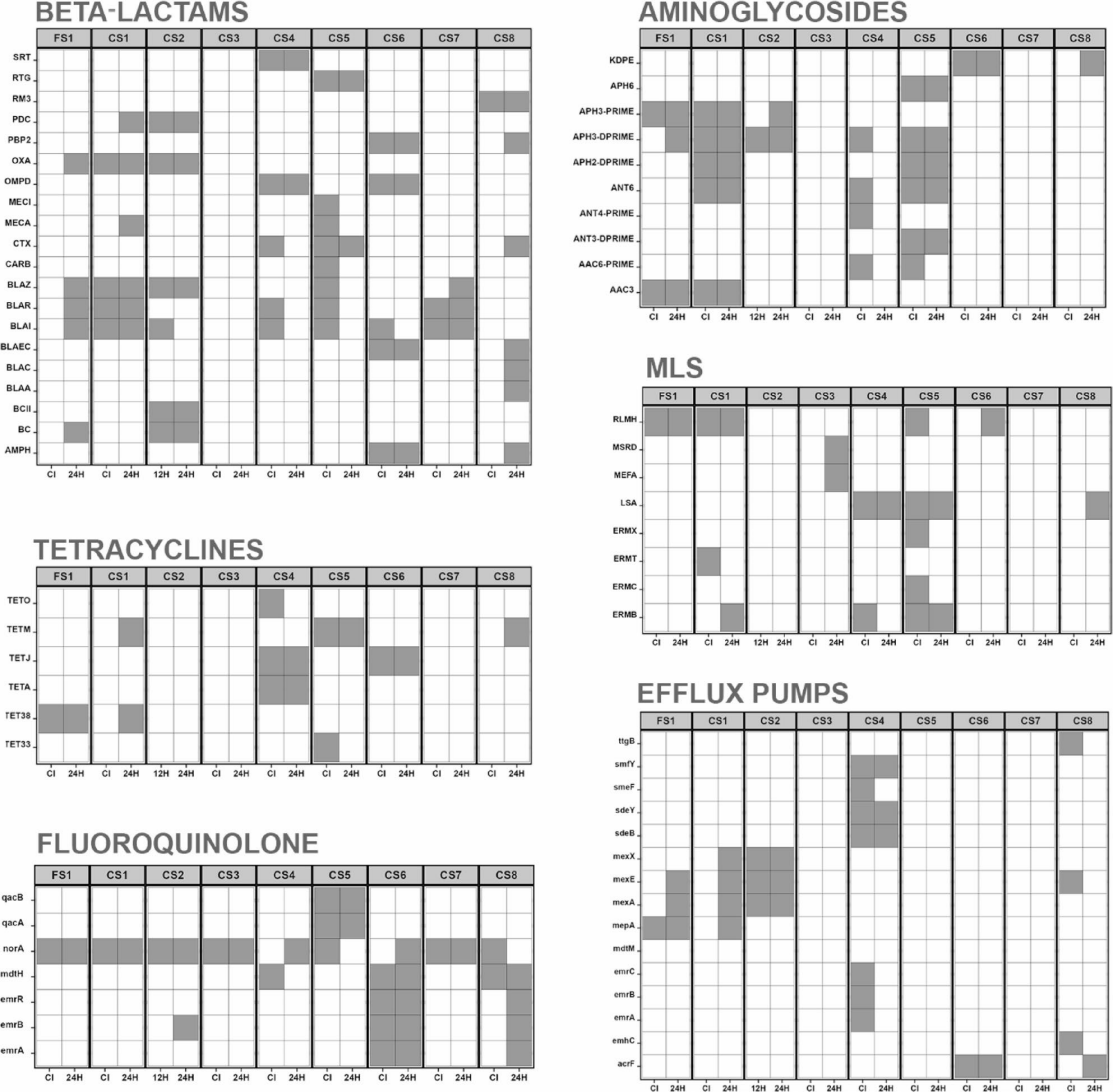
MGX Resistome: Beta-lactam, aminoglycoside, fluoroquinolone, macrolide, lincosamide, streptogramin (MLS), tetracycline, efflux pump, observed in feline and canine skin samples Patient numbers (i.e.; FS1 = Feline Skin 1, CS1 = Canine skin 1, etc.) are listed across the top of each panel with culture independent (CI) metagenomic or quasi-metagenomic enriched data type (24 h) identified along the x axis. Presence/absence of MGX detected ARGs from beta-lactam, aminoglycoside, tetracycline, macrolide, lincosamide, streptogramin (MLS), fluoroquinolone, and efflux pump classes are shown. ‘Presence’ is considered a fully covered gene. Numbers of sequences hitting individual genes (grey boxes) span 1 to 51,000

**Fig. 3:**
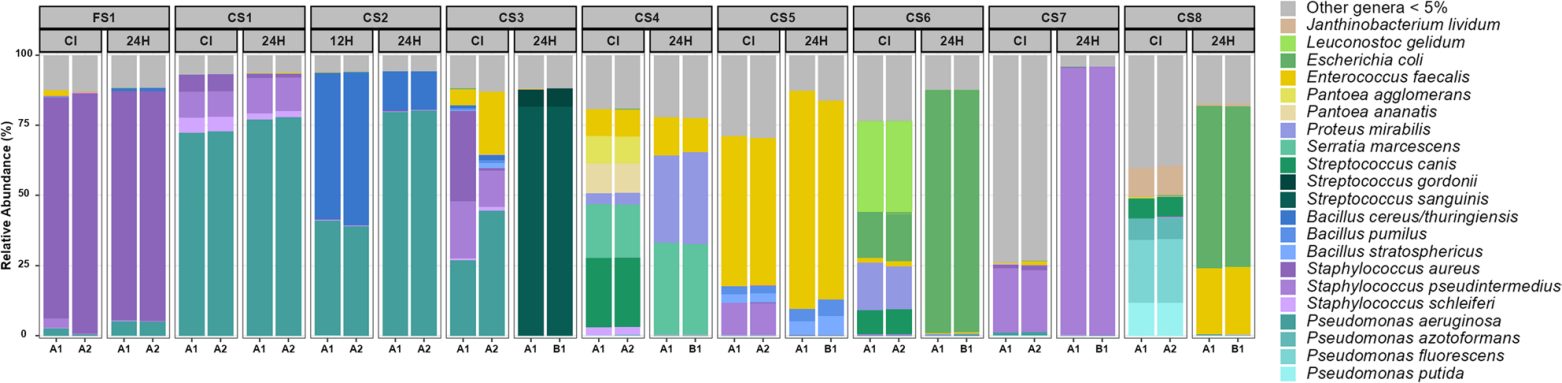
Bacterial microbiomes of skin infection samples Relative abundance of bacterial taxa are shown for culture independent (CI) and quasi-metagenomic enriched data (24 h). Other genera present below 5% relative abundance are designated with grey. Two technical replicates are shown for each data type

**Fig. 4:**
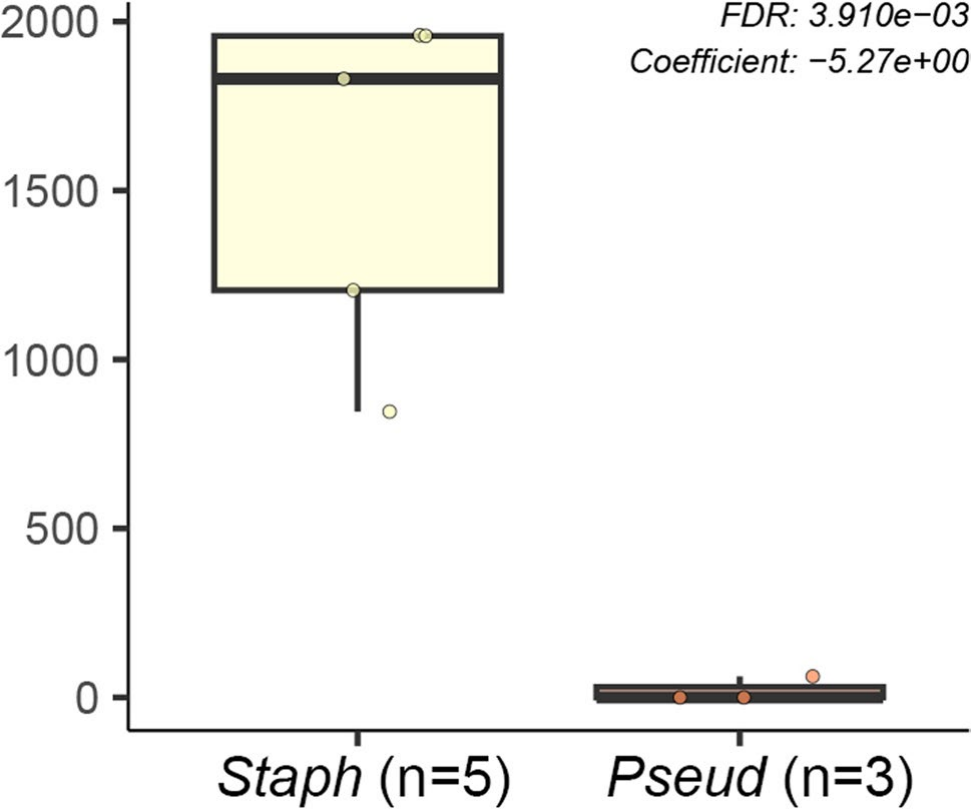
4-deoxy-l’threo-hex-4-enopyranuronate is differentially abundant in skin samples from which *Staphylococcus* or *Pseudomonas* were recovered The Microbiome Multivariate Association with Linear Models (MaAsLin) pipeline, was used to identify a statistically significant increase in metabolic feature: 4-deoxy-l’threo-hex-4-enopyranuronate in samples from which *Staphylococcus* was recovered, contrasted to *Pseudomonas*

**Fig. 5:**
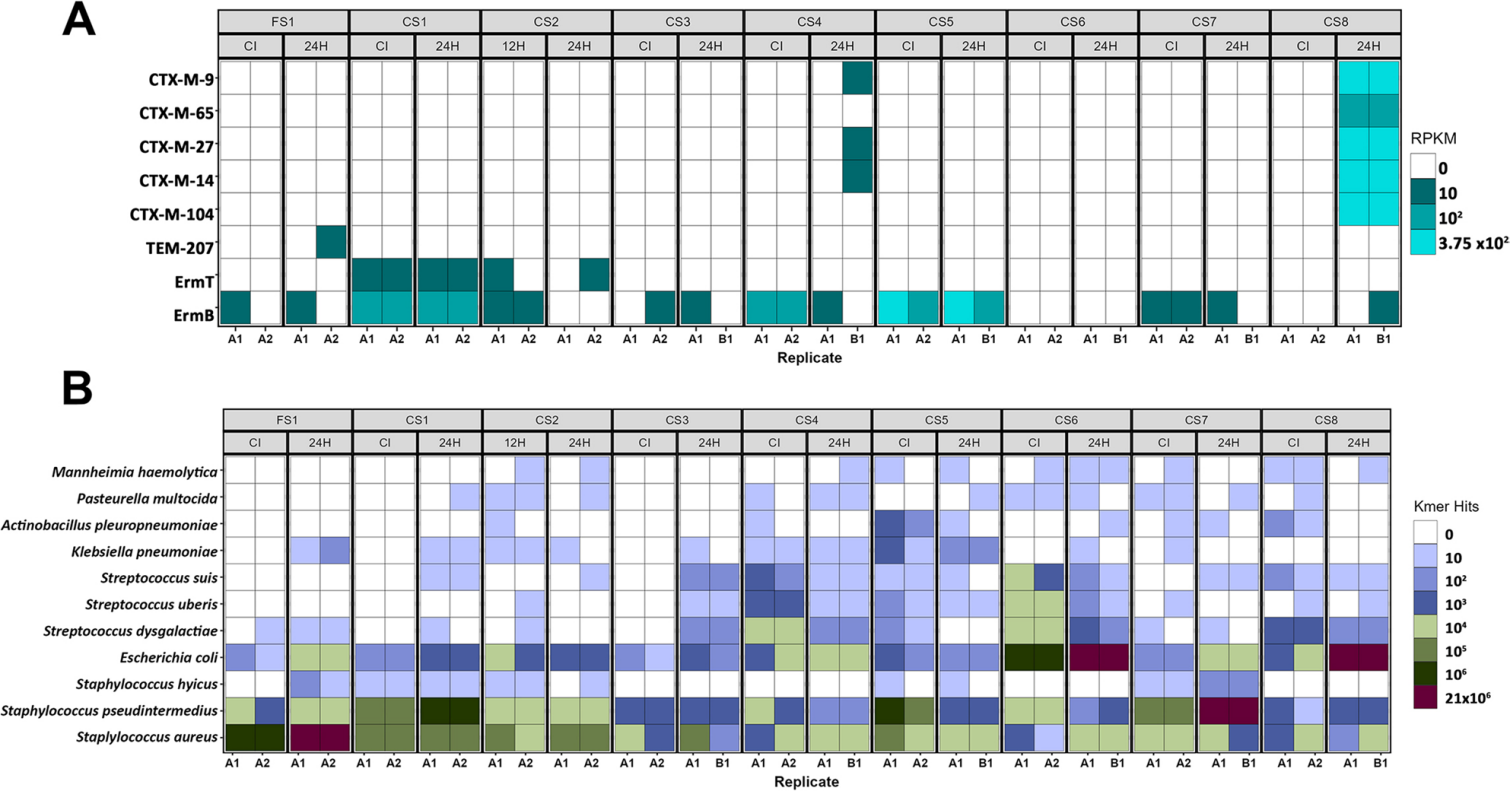
NARMS ARG monitoring targets and EARS-Vet Pathogen monitoring targets observed in feline and canine skin samples **A**. NARMS monitoring ARG targets observed across canine and feline infection samples and (**B**). EARSVet pathogens observed in skin infection samples for MGX (CI) and enriched quasi-metagenomic data (24 h)

MGX data detected the same pathogens recovered by culture methods (Fig. 1) and simultaneously described antimicrobial resistance genes (ARGs) (Figs. 2 and 3), plasmid markers, multi-domain microbiota including bacteria (Fig. 4), viruses and phages, fungi and protists, and metabolic features (Fig. 5). In cases where VDL AST recovered methicillin resistant isolates, metagenomic data identified methicillin resistance gene determinants. *Mec*A was observed in MGX data for sample CS4 (VDL AST recovered *MR Staphylococcus schleiferi)*, and CS8 (VDL AST recovered *Staphylococcus schleiferi)* and *mec*A, *mec*R1, and *mec*I were observed in CS5 (VDL AST recovered *Staphylococcus pseudintermedius)* (Fig. 1). Metagenomic and quasi-metagenomic data also detected *mec* genes in four other case samples for which no MR isolates were recovered by VDL AST (Fig. 1).

### Resistome

An overview of antimicrobial resistance genes observed in MGX data are shown for each sample in Fig. 2. Multiple AGRs across classes of critically important antimicrobial resistance genes were identified by MGX data including beta-lactam genes: CTX-M-104, CTX-M-14, CTX-M-27, CTX-M-65, and CTX-M-9, TEM 207, aminoglycosides, fluoroquinolones, macrolides, lincosamides, streptogramins, tetracyclines, efflux pumps, (Fig. 2) sulfonamides, and tellurium resistance.

Trimethoprim–Sulfamethoxazole (folate inhibitors) resistance genes: *sul1* and *sul2* were both observed in CS5, and *sul2* was observed in sample CS6. The *terZ* (tellurium resistance) was observed in CS4 and CS6.

### Assemblies

MGX and qMGX data were assembled to create contigs to potentially link AMR to plasmids and/or chromosomes. In sample, CS1, a contig of 112,255 bp carrying *mecA* was assembled with a 99.77% BLAST match to *Staphylococcus coagulans* strain 1,031,371. In sample CS2, another, much shorter contig (2119 bp) with *mecA* also had a 99.86% BLAST match to *Staph. coagulans* 1,031,371 (Table 2).

**Table 2:** Taxonomic assignment of contigs carrying *mecA* genes

sample	% id	query length	alignment length	coverage	evalue	bit score	taxonomic assignment
CS1	99.77	112,246	105,573	0.94	0	104,823	*Staphylococcus coagulans* strain 1,031,373 chromosome
CS2	99.86	2119	2119	1.00	0	2110	*Staphylococcus coagulans* strain 1,031,373 chromosome

### Plasmids

Plasmids were annotated in five of the nine samples using assembled contigs with Platon [26]. A summary of the replicon types detected in MGX data from feline and canine skin samples is summarized below with full details by sample and associated resistance in Table 3.

- FS1: IncI1, IncI2, IncP, IncA/C, IncA/C-like, IncFII(pKPX-1), Inc18.
- CS1: pSK41-derivative (integrated).
- CS4: pSK41 (Gram-positive).
- CS5: RepUS11, RepUS14 (mobilizable).
- CS8: IncFII, IncFIB.

**Table 3:** Replicon families of plasmids identified in skin samples with associated drug resistance classes

Skin ID	Replicon Family	Drug Resistance Classes
FS1	IncI2	Polymyxins (mcr-1)
FS1	IncA/C-like	AmpC Beta-lactams (CMY-2), Phenicols, Sulfonamides, Tetracyclines
FS1	IncA/C	AmpC Beta-lactams (CMY-2), Phenicols, Sulfonamides, Tetracyclines
FS1	IncI1	Tetracyclines, Streptomycin, Sulfonamides
FS1	IncFII(pKPX-1)	Carbapenems (KPC-2)
FS1	IncP	Polymyxins (mcr-3), Carbapenems (NDM-5)
FS1	Inc18	Glycopeptides (vanA), Aminoglycosides, Macrolides
CS1	pSK41-derivative (integrated)	Trimethoprim, Tetracyclines, MLS_B
CS4	pSK41 (Gram-positive)	Aminoglycosides, MLS_B, Tetracyclines, Cadmium
CS5	RepUS11	Oxazolidinones (linezolid), Cytolysin
CS5	RepUS14 (mobilizable)	Tetracyclines
CS8	IncFII	Extended-spectrum Beta-lactams (CTX-M)
CS8	IncFIB	Extended-spectrum Beta-lactams (CTX-M-15), Fluoroquinolone efflux (oqxAB)

### Bacterial microbiomes

Other dominant Gram-negative species observed across the samples in metagenomic data included *Serratia marcescens*,* Pantoea ananatis*,* Pantoea agglomerans* (CS4), and *Escherichia coli* (CS6) as well as additional *Pseudomonas* species (Fig. 3).

Many *Pseudomonas* species were detected in MGX data. The ten most abundant species included *Pseudomonas aeruginosa*, *P. azotoformans*,* P. fluorescens*, *P. rhodesiae*,* P. coleopterorum*,* P.putida*,* P. protegens*,* P. proteolytica*,* P. trivialis*, and *P. extremorientalis* (Table 4).

**Table 4:** Ten most abundant *Pseudomonas* species in metagenomic data of skin samples

Pseudomonasspecies	FS1	CS1	CS2	CS3	CS4	CS5	CS6	CS7	CS8
*Pseudomonas aeruginosa*	4771	1,443,264	3,992,762	13,285	4908	10,290	2864	6350	3272
*Pseudomonas azotoformans*	1	3	108	0	50	266	3	1	154,989
*Pseudomonas fluorescens*	1	12	151	0	927	148	6	184	148,889
*Pseudomonas rhodesiae*	0	2	82	0	65	1	3	0	121,802
*Pseudomonas coleopterorum*	10	3	15	0	62,550	9	4	0	15,897
*Pseudomonas putida*	1	14	127	0	366	21	2	178	67,817
*Pseudomonas protegens*	0	2	39	0	54	3	0	0	52,601
*Pseudomonas proteolytica*	0	0	14	0	29	15	1	0	20,634
*Pseudomonas trivialis*	0	1	20	0	140	8	1	2	15,129
*Pseudomonas extremorientalis*	0	1	89	0	37	1	0	1	10,641

*Staphylococcus pseudintermedius* was recovered by VDL culture-based testing for three infection samples: CS5, CS6, and CS7. In MGX data, *S. pseudintermedius* was observed in every sample however in 4 samples (CS2, CS4, CS6, and CS8), its relative abundance was under 1% (Fig. 1). *Staphylococcus schleiferi* was recovered by VDL culture-based testing for three canine infections. It was also detected in every metagenomic dataset however in low abundance (under 1% for six of the nine samples: FS1, CS2, CS5, CS6, CS7and CS8). For sample CS6, *S. schleiferi* is not shown in Fig. 1 because its abundance was so low (0.000722). In two samples from which VDL culture recovered *S. schleiferi*, (CS1 and CS4) *its* abundance in metagenomic data was 5.36 and 2.88 respectively. The sample (CS8) from which *S. schleiferi* was recovered by VDL had only 0.03 abundance of that taxon in the metagenomic data.

Multiple *Staphylococcus* species co-occurred in every infection microbiome, primarily *S. aureus*,* S. pseudintermedius*,* S*. *schleiferi* and *S. saprophyticus*. Additional *Staphylococcus* species observed in low abundance included *S. capitis*,* S. epidermidis*,* S. hyicus*,* S. pasteuri*,* S. simulans*,* S. succinus*,* S. virulinus*,* S. warneri*, and *S. xylosus*. A complete list of all bacterial species and their relative abundance across infection samples is available in Supplementary Materials (S2: Skin bactikmer RA AVGreps CI 1%).

### Eukaryotic taxa: fungal and protozoan

The fungal pathogen *Malasseszia pachydermatis* was observed in samples CS1, CS3, and CS8 (very low abundance in CS3) and a very small number of hits to *Malassezia restricta* were observed in the feline sample FS1. Other fungal taxa observed in MGX data included *Mucor racemosus*, and *M. plumbeus*, detected in high abundance in sample CS6. Protozoan species *Cytauxzoon felis* was observed exclusively in the feline sample FS1 and *Acanthamoeba palestinensis* was observed in CS3, and CS4. Full details by relative abundance are available in Supplementary Materials (S3: Eukaryotic_Viral_Skin).

### Viruses and phages

Metagenomic data detected multiple virus and phage species in feline and canine skin samples. Torque teno canis virus was observed in canine sample CS6 and Gammaretrovirus RD114 was observed in the feline sample FS1. Also observed exclusively in the feline sample was Feline leukemia virus. The most prevalent phage was *Pseudomonas* phage Pf1, observed across all samples in correlation with prevalence of *Pseudomonas*. A full list of the relative abundance of viral, phage, fungal and protozoan species is available in Supplementary Materials (S3: Eukaryotic_Viral_Skin).

### Functional gene data

Only one significant differentially abundant metabolic feature was identified when contrasting samples from which *Staphylococcus or Pseudomonas* were recovered. Genes involved in production of uronic acid (4-deoxy-l’threo-hex-4-enopyranuronate) were significantly elevated in canine infections from which *Staphylococcus* was recovered contrasted to infections from which *Pseudomonas* was recovered (Fig. 4).

### Monitoring Targets

Clinically important AMR genes monitored by the National Antimicrobial Resistance Monitoring System (NARMS) were observed across metagenomic and quasi-metagenomic data (Fig. 5A). Pathogens monitored by the European Antimicrobial Resistance Surveillance Network in Veterinary Medicine (EARS-Vet) include: *Escherichia coli*,* Klebsiella pneumoniae*,* Mannheimia haemolytica*,* Pasteurella multocida*,* Actinobacillus pleuropneumoniae*,* Staphylococcus aureus*,* Staphylococcus pseudintermedius*,* Staphylococcus hyicus*,* Streptococcus uberis*,* Streptococcus dysgalactiae*,* and Streptococcus suis.* Taxa from that surveillance program observed in metagenomic and quasimetagenomic data from canine and feline infection samples are shown in Fig. 5B.

### ML based predictions

A logistic regression model to predict methicillin resistance using relative abundance of the top 32 bacterial species (excluding the four pathogens: *Pseudomonas aeruginosa*,* Staphylococcus schleifleri*,* Staphylococcus pseudintermedius*, and *Staphylococcus aureus*) was tested for its ability to accurately predict recovery of a methicillin resistant phenotype. Results showed correlative prediction, however biological relevance is limited due to the small sample size. A decision tree model to delineate cutoff abundances for one or more species that would differentiate methicillin resistant infections produced an over simplified tree with only one split: classifying all samples with > 0.013 abundance of *Pantoea agglomerans* as having MR strains. Only the FS1 (feline) sample did not follow the pattern so there was not 100% agreement. Lastly, a random forest model (collection of decision trees) was evaluated for its accuracy to predict methicillin resistance. Initial tests suggested that 40 trees were optimal with prediction scores of 100%. For all models, however, prediction success was likely due to the small test set and as such primarily represents a future use case for MGX data in veterinary diagnostics that may, when conducted with more rigorous experimental design, (beyond what was possible with a small case study), provide valuable hypotheses and identify clinically significant trends.

## Discussion

Previous culture based veterinary diagnostics described *Staphylococcus pseudintermedius* as the most frequently recovered Gram-positive species from canine skin infections [27]. In the case series examined here, we observed equal recovery of *Staph*. *pseudintermedius*, and *Staph schleiferi*. For gram negative species associated with canine skin infections, *Pseudomonas aeruginosa* has been described as the most frequently recovered pathogen [27] which aligned with the Gram-negative pathogen recovery observed in this case series. Additional work will be needed to more comprehensively understand concordance of culture-based and MGX methodologies.

### Resistome

Monitoring AMR and plasmids provides insight into the total resistome potential of an environment or sample matrix. The IncI, IncI2, IncA/C, IncF and IncP plasmid families (Table 5) observed in metagenomic data from this case series are well known Gram-negative AMR vectors across bacterial species associated with humans and animals. The pSK41-family plasmids are Gram-positive conjugative multi-resistance replicons in Staphylococci and the pheromone-responsive RepUS11 plasmids (observed in sample CS5) are a growing concern because they connect linezolid resistance with high-level virulence in *Enterococcus* [28]. *Enterococcus faecalis* was observed in every infection sample.

**Table 5:** Plasmid types observed in metagenomic data and associated resistance mechanisms

Plasmid Type:	Observed in Samples:	Primary Resistance Mechanisms:
IncFII/IncFIB	FS1, CS8	optrA/poxtA (linezolid), TMP/sulfa resistance
pSK41-related	CS1, CS4	MLS_B, tetracycline, aminoglycoside resistance
Multiple Inc types	FS1	Broad-spectrum resistance including tet, cat, arr genes

The simultaneous identification of pathogens (Fig. 1), total microbial ecology (Fig. 3), and total resistomes (Fig. 2), may, when appropriately optimized and validated, direct more precise and judicious antimicrobial treatment. As proof of concept for this idea, we used VDL results with internal FDA artificial intelligence (AI) ELSA to ask what antibiotic course of action would be recommended for treatment of infections based on VDL recovered pathogens. We then asked if antibiotic prescription should be adjusted based on the additional information provided by MGX data. Adjusted suggestions were made for seven of the nine samples based on genes and taxa identified in MGX data. For example, for FS1, the original AI based suggestion was use clindamycin or amoxicillin-clavulanate. With the addition of MGX data describing blaZ and FosB genes, the adjusted AI suggestion was to avoid beta-lactams and fosfomycin in treatment. A full list of metagenomic informed antibiotic treatment suggestions and MGX data-based adjustments to those suggestions is presented in Supplementary Materials (S4: AI suggested antibiotic treatments should be regarded as research and not taken as clinical treatment recommendations).

### Cross domain insight

The polymicrobial profiles that metagenomic data provide, underpin potential identification of cross domain linkage, co-occurrence, and biological relationships that may improve our understanding of currently uncharacterized infection ecologies and provide new treatment strategies. In the past, leveraging information about cross domain relationships supported successful treatments for heartworm (*Dirofilaria immitis*) in dogs by treating the bacterial symbiont *Wolbachia* with doxycycline [29, 30] and for lymphatic filariasis in humans, where elimination of *Wolbachia* from the eukaryotic parasite *Wucheria bancrofti* was an effective treatment for parasitic infections [31–33]. MGX data may also be useful for improved understanding of prevalence and co-occurrence of zoonotic pathogens. Methicillin-resistant staphylococci (MRSA, MRSP, MRSS) can transfer between humans and pets making co-occurrence of these species in feline and canine infections important to understand. The detection of retrovirus RD1114 may also be significant for One Health considerations because RD1114 unlike typical endogenous retroviruses that are transmitted vertically through germline integration, has demonstrated capacity for horizontal transmission between species.

*Alternaria*, *Malassezia*,* and Aspergillus* have been described as the most commonly observed fungal genera associated with allergy and infection in animals [34]. *Malassezia* was well documented in MGX data from this case series, but few other fungal species besides *Mucor* spp. were identified. A dynamic and at times antagonistic interplay between *Malassezia* and *Staphylococcu*s has been described, which reportedly led to reduced susceptibility to azole antifungals for *Malassezia* when co-cultured with *Staphylococcus* [35, 36]. Co-occurrence and enrichment of both taxa has been observed in scalp microbiomes of human patients with seborrheic dermatitis [37] and *Malessezia* has been shown to antagonize *Staphylococcus* and disrupt its biofilm formation [38]. A symbiotic relationship between *Malassezia pachydermatis* and commensal staphylococci has also been proposed [39]. *Malassezia* and *Staphylococcus* co-occurred in four infections from this case series. Perhaps intentional manipulation of *Malassezia* could impact survival of *Staphylococcus* in MR infections, providing new approaches when antibacterials are not effective.

### Biomarker identification

Additionally, we highlight the potential for MGX data to support metabolomic or chemical biomarker discovery. Metabolomic data have been used to differentiate between feline chronic enteropathy and small-cell lymphoma [40], as well as to provide biomarkers for canine sepsis [41], sow pregnancy [42], and hyperketonaemia in dairy cows, to list a few. Metabolomic screens are expensive and complicated and if DNA data can be used to identify differential incidence of key metabolite pathways, typically measured by chemical techniques, this could provide a rapid and less costly approach to identification of novel diagnostic features [43, 44]. The accessibility of biomarkers for diagnostic etiologies, features, or disease states based on functional metabolic genes is rapidly changing the way in which patients are evaluated and managed for certain conditions. If specific biomarkers could be linked to *Staphylococcus* infections, for example, to distinguish them from *Pseudomonas* infections, that could potentially expedite initial treatments.

### Monitoring

A benefit of metagenomic data is the ability to contribute to numerous objectives with the same dataset. MGX Data from skin infections was useful for pathogen detection and for broader monitoring efforts. The National Antimicrobial Resistance Monitoring System (NARMS) of the Center for Veterinary Medicine at the FDA in collaboration with the Veterinary Laboratory Investigation and Response Network (Vet-LIRN) actively monitors a subset of colistin, beta-lactam, fluoroquinolone, and macrolide resistance determinants in *Salmonella*,* Escherichia coli*,* Campylobacter*, *Enterococcus*,* Klebsiella*,* Aeromonas*,* and Vibrio.* The European Antimicrobial Resistance Surveillance Network in Veterinary Medicine (EARS-Vet) [45], (38 partners spanning 18 countries) coordinates with the World Health Organization (WHO) to monitor *Escherichia coli*,* Klebsiella pneumoniae*,* Mannheimia haemolytica*,* Pasteurella multocida*,* Actinobacillus pleuropneumoniae*,* Staphylococcus aureus*,* Staphylococcus pseudintermedius*,* Staphylococcus hyicus*,* Streptococcus uberis*,* Streptococcus dysgalactiae*,* and Streptococcus suis.* MGX data provided proof of concept that broader surveillance interests can be addressed with the same data used to detect specific pathogens.

### Limitations

While many data, without appropriate standards for quality and interpretation can actually confound rather than improve treatment [46], the integration of MXG data into veterinary diagnostics has demonstrated utility to support monitoring efforts, to provide broader understanding of co-occurring species, infection ecologies, transmission resistance potential, zoonotic risk assessment, polymicrobial epidemiology, and ML-based predictions. Proof of concept is achieved for many of those aims but biological inference may be limited due to the small number of samples examined in this study. The ML models (linear regression, decision tree, and random forest) that were run using metagenomic and quasi-metagenomic input from infection samples demonstrated proof of concept for actionable ML applications to advance veterinary diagnostics, however, they all fell short of reliable biological inference due to the small number of samples. Discordance of the feline sample (FS1) from agreement observed for canine samples in the decision tree model to predict methicillin resistance using a species-specific cut off, supports the idea that species specific microbiome informed models might, with the right sample size, effectively predict resistant phenotypes.

Another limitation of short read MGX sequence data is confidence regarding both identification of specific taxa and linkage of antimicrobial resistance determinants to specific pathogenic or non-pathogenic taxa. With short sequence reads, highly accurate annotations of taxa and genes are possible, but challenges exist depending on the diversity of the environment, sequencing depth, closely related lineages, accurate references, and other factors. The high-quality metagenome assembled genomes (MAGS) assembled from this dataset are one way of addressing resolution issues from MGX and qMGX data. Two of the high-quality MAGS carried the *mecA* gene and could be aligned with high confidence to *Staphylococcus coagulans* in CS1 and CS2, (Table 2) which was not identified by VDL results. This discordance highlights the utility that synchronous application of VDL, WGS, and MGX approaches can bring to the continually advancing frontier of precision veterinary diagnostics. The fact that multiple pathogens were seen in every infection suggests there may be greater complexity to the skin infections evaluated here than a single VDL AST isolate can explain, and that diverse data types used collaboratively may advance our understanding of potentially polymicrobial etiologies, effectively underpinning more targeted strategies for precision antimicrobial stewardship.

### Accelerated cures

A primary objective for FDA is the acceleration of cures [47] for the American public. For the Center for Veterinary Medicine (CVM), American companion animals are part of a One Health research focus. Cures and treatment cannot be expedited if diagnostics do not sufficiently inform medical response. The case study presented here highlights a frontier of opportunity for advancing veterinary diagnostics using MGX data. Simultaneous identification of pathogens, co-occurring species, cross domain linkages, ARGs, plasmids, and metabolic features could improve diagnostics for complex states of disease while simultaneously stewarding antimicrobial resistance. Additionally, MGX data input to ML models shows promise for advancing new diagnostic approaches. In this case study, we evaluated metagenomic data in coordination with state-of-the-art VDL aerobic culture and AST results for a case series of feline and canine skin samples. Results provide numerous insights into opportunities to advance One Health approaches to veterinary diagnostics.

## Materials and methods

### DNA Extraction

Skin was swabbed by a veterinarian with Amies non charcoal swabs and directly plated to blood, MacConkey and Columbia CNA plates. Swabs were stored at -20°at UPenn Veterinary Hospital and shipped to FDA Center for Veterinary Medicine labs for pilot MGX analyses. Swabs were halved and DNA was extracted from one half that received no enrichment and DNA from the second half was extracted after 24 h enrichment in universal pre-enrichment broth (UPB) at 37° using the Qiagen DNeasy Blood and Tissue Kit according to the manufacturer’s specifications. CI (Culture Independent) refers to metagenomic (MGX) samples and samples enriched for 12, or 24 h are considered quasimetagenomic (qMGX). A minimum of two technical replicates of MGX and qMGX were used for each skin sample. Replicates of each type were merged for certain summaries and/or visualizations.

### Library preparation and sequencing

DNA libraries were prepared using the Illumina DNA Library Prep Kit according to the manufacturers specifications (Illumina).

https://www.protocols.io/edit/illumina-dna-prep-sop-bzstp6en.

Sequencing was performed on a NextSeq 2000 with 2 × 150 cycles using the NextSeq 1000/2000 P2 Reagent Kit (300 Cycles). Libraries were diluted to a 750 pM loading concentration according to the Illumina’s specifications (NextSeq Denature and Dilute Libraries Guide*) *https://support.illumina.com/content/dam/illumina-support/documents/documentation/system_documentation/NextSeq2000/nextseq-1000-2000-sequencing-system-guide-1000000109376-05.pdf.

### Bioinformatic analysis

Sequencing data was demultiplexed (bcl2fastq2), screened/trimmed using Trimmomatic [48]. Quality-checked reads resulted in an average of 22 million reads per sample for further downstream analyses.

### AMR and plasmid annotation

Paired end FASTQ files were analyzed using the AMR + + pipeline [49] with the MEGARes database v2 using default parameters. Additionally, the AMRFinder Plus database was used for AMR annotation using SAUTE [50] on the FDA Human Foods Program (HFP) high performance cluster(HPC). https://github.com/ncbi/amr/wiki/Methods and BLAST was used with the CARD [51] database to annotate sequence data according to default parameters. Reads were also evaluated using the COSMOS ID analytical pipeline (AMR database update May 2025 https://www.cosmosid.com ) to contrast with in-house results). Counts and abundances from AMR annotation outputs were ‘normalized’ using scripts to assess ‘reads per kilobase million’ (RPKM) to normalize gene reporting between different sites by accommodating for variation in number of sequencing reads per sample and gene length. Total reads in the sample were divided by 1,000,000 “per million” scaling factor to normalize for sequencing depth and provide ‘reads per million’ (RPM). RPM values were then divided by length of each gene in kilobases to report ‘RPKM. https://github.com/SethCommichaux/AMRplusplus. Identification of plasmids was accomplished using assemblies by MEGAHIT [52] and Platon [26] for plasmid replicon identification and BLAST NCBI tools [53] for contig taxon assignment.

### Bacterial annotation

Determination of bacterial composition from shotgun sequencing was conducted using custom C + + compiled *k*-mer signature databases containing multiple unique 30 bp sequences per species evaluating each read with 30 bp probes. This FDA in-house bacterial *k*-mer database (Bactikmer) contains ~ 5900 target taxonomic entries, each consisting of approximately 40,000 (range 44 to 80,000) unique *k*-mers. The database includes 1100 different bacterial genera, and 3500 species – pathogens important to FDA food safety queries and close relatives of those taxa for clear species delineation (GitHub - mmammel8/kmer_id: Identification of WGS reads by kmer database). Normalization is performed to correct for bias due to differing number of *k*-mers used per database entry and results are tabulated as percentage of identified reads for each database entry. Kraken [54] is also used as a first pass taxonomic classifier.

### Fungal, protist, virus, phage, and metabolic feature annotation

The same method described above was created to annotate fungal, protozoa, and viral taxa. Reads from annotations were double-checked by Blasting them against genomes of identified organisms to evaluate accuracy of k-mer based annotation. Additionally, the COSMOS ID analytical pipeline with fungal database was used to contrast with results from the FDA in-house fungal annotation pipeline and database (https://www.cosmosid.com).

For functional gene/metabolic feature annotation and evaluation the Microbiome Multivariate Association with Linear Models (MaAsLin) pipeline from the Huttenhower biobakery tools (GitHub - biobakery/Maaslin2: MaAsLin2: Microbiome Multivariate Association with Linear Models) was used in the COSMIS ID application to identify differentially observed metabolic features (www.cosmosid.com).

### Machine Learning (ML) modeling

To predict methicillin resistance, the top 32 species (excluding MR species) were used as input data. The data were split into training and test sets. 70% of the data were used to train logistic regression models and the other 30% were tested to see if the model would correctly predict on held out data. 100% concordance was achieved on the limited sample size.

A decision tree model was also explored to find cutoff abundances in one or more species that would split the samples by resistance prediction. One tree was created with one split at 0.013 abundance of *Pantoea agglomerans*. The model accurately classified all canine samples with greater than 0.013 abundance of *Pantoea agglomerans* as having MR strains. The feline sample did not follow the pattern. A random forest (collection of decision trees) model suggested the number of trees to use was 40. Prediction scores were 100% but the small sample size makes biological inference limited. A python notebook for this work is provided in the Supplementary Materials (S5: Supplementary Python Notebook).

## Data reporting and visualization

Pipeline annotation outputs were visualized using R Studio (version 4.5.0). Visualizations were created using ggplot2 [55] and Adobe Photoshop.

## Supplementary Information

Supplementary Material 1.

Supplementary Material 2.

Supplementary Material 3.

Supplementary Material 4.

Supplementary Material 5.

## Data Availability

The data presented here have been deposited at the National Center for Biological Information (NCBI) under BioProject PRJNA1335144 with accession numbers SAMN51891460 through SAMN51891495 and SRA accession numbers SRR35632839 through SRR35632874. Metadata follow the Genomic Standards Consortium (GSC) Minimum Information about any Sequence (MIxS) compliant MIMS metagenome/environmental host associated package (version 6). All data are available here: [https://www.ncbi.nlm.nih.gov/bioproject/PRJNA1335144/](https:/www.ncbi.nlm.nih.gov/bioproject/PRJNA1335144).

## Abbreviations

AMR: Antimicrobial resistance
ARG: Antimicrobial resistant gene
AST: Antimicrobial susceptibility testing
DTR: Difficult to treat resistance
EARS-Vet: European Antimicrobial Resistance Surveillance Network in Veterinary Medicine
FDA: Food and Drug Administration
HGT: Horizontal gene transfer
MGX: Metagenomic
MDR: Multi–drug resistant
MRSP: Methicillin resistant *Staphylococcus pseudintermedius*
MR: Methicillin resistant
MRSS: Methicillin resistant *Staphylococcus schleiferi*
NARMS: National Antimicrobial Resistance Monitoring System
NGS: Next generation sequencing
PCR: Polymerase chain reaction
qMGX: Quasimetagenomics, referring to shotgun sequencing of enrichments
VDL: Veterinary diagnostic laboratories
WHO: World Health Organization
