# Supplementary material for: Breaking the culture habit: Complementing culture-based veterinary diagnostics with metagenomic data -A case study of feline and canine skin infections
Source: BMC Vet Res. 2026 May 9;22:362. doi: 10.1186/s12917-026-05476-x (PMC13285402; doi:10.1186/s12917-026-05476-x)
Supplement: Supplementary file 3 — Supplementary Material 3. [file 12917_2026_5476_MOESM3_ESM.pdf]

dogskin

January 11, 2024

[2]:
```
import os
import pandas as pd
import numpy as np
import seaborn as sns
import matplotlib.pyplot as plt
from sklearn import datasets
import math
%matplotlib inline
```

1 Data: relative abundance

1.1 First look at data

[4]:
```
#load dataset into Pandas
filepath = "dogskin_b_tr.csv"
data = pd.read_csv(filepath)
data.head()
```

[4]:
```
          name  C_Pseudomonas_aeruginosa  C_Staphylococcus_schleiferi  \
0  S1_OH_CI_A1                         1                            1
1  S1_OH_CI_A2                         0                            0
2  S3_OH_CI_A1                         0                            0
3  S3_OH_CI_A2                         0                            0
4  S5_OH_CI_A1                         1                            0

   C_Staphylococcus_pseudintermedius  C_Staphylococcus_aureus  C_MR  \
0                                  0                        0     0
1                                  0                        0     0
2                                  0                        1     0
3                                  0                        1     0
4                                  0                        0     0

   Enterococcus_faecalis  Serratia_marcescens  Leuconostoc_gelidum  \
0                  0.010                0.000                  0.0
1                  0.062                0.000                  0.0
2                  1.708                0.000                  0.0
3                  0.150                0.488                  0.0
```

```
4                  4.576                0.000                  0.0

   Janthinobacterium_lividum  …  Pantoea_septica  Pseudomonas_marginalis  \
0                        0.0  …            0.000                     0.0
1                        0.0  …            0.002                     0.0
2                        0.0  …            0.097                     0.0
3                        0.0  …            0.006                     0.0
4                        0.0  …            0.239                     0.0

   Corynebacterium  Agromyces_italicus  Xylanimonas_cellulosilytica  \
0            0.000                 0.0                        0.000
1            0.000                 0.0                        0.000
2            0.004                 0.0                        0.042
3            0.002                 0.0                        0.000
4            0.000                 0.0                        0.000

   Pseudomonas_trivialis  Bacillus_cereus/thuringiensis  Bacillus_pumilus  \
0                    0.0                          0.012             0.000
1                    0.0                          0.013             0.000
2                    0.0                          0.002             0.061
3                    0.0                          0.033             0.000
4                    0.0                          1.072             0.000

   Streptococcus_sanguinis  Streptococcus_gordonii
0                    0.038                   0.000
1                    0.000                   0.000
2                    0.298                   0.003
3                    0.214                   0.001
4                    0.000                   0.000

[5 rows x 38 columns]
```

[5]:
```
data.describe()
```

[5]:
```
       C_Pseudomonas_aeruginosa  C_Staphylococcus_schleiferi  \
count                 16.000000                    16.000000
mean                   0.187500                     0.312500
std                    0.403113                     0.478714
min                    0.000000                     0.000000
25%                    0.000000                     0.000000
50%                    0.000000                     0.000000
75%                    0.000000                     1.000000
max                    1.000000                     1.000000

       C_Staphylococcus_pseudintermedius  C_Staphylococcus_aureus    C_MR  \
count                             16.000                16.000000  16.000
mean                               0.375                 0.125000   0.375
```

std	0.500	0.341565	0.500
min	0.000	0.000000	0.000
25%	0.000	0.000000	0.000
50%	0.000	0.000000	0.000
75%	1.000	0.000000	1.000
max	1.000	1.000000	1.000

	Enterococcus_faecalis	Serratia_marcescens	Leuconostoc_gelidum	\
count	16.000000	16.000000	16.000000	
mean	8.070125	6.715562	5.415312	
std	14.574874	18.223564	14.799161	
min	0.010000	0.000000	0.000000	
25%	0.141500	0.000000	0.000000	
50%	1.897000	0.000500	0.000000	
75%	6.095000	0.064000	0.002250	
max	43.862000	53.635000	44.219000	

	Janthinobacterium_lividum	Proteus_mirabilis	…	Pantoea_septica	\
count	16.000000	16.000000	…	16.000000	
mean	3.541000	3.354188	…	0.466125	
std	9.663298	8.073480	…	1.031493	
min	0.000000	0.000000	…	0.000000	
25%	0.000000	0.000000	…	0.001750	
50%	0.000000	0.003000	…	0.007000	
75%	0.008000	0.734500	…	0.132500	
max	28.430000	25.105000	…	3.053000	

	Pseudomonas_marginalis	Corynebacterium	Agromyces_italicus	\
count	16.000000	16.000000	16.000000	
mean	0.398438	0.395250	0.384312	
std	1.088405	0.855761	0.959828	
min	0.000000	0.000000	0.000000	
25%	0.000000	0.000000	0.000000	
50%	0.000000	0.034500	0.007000	
75%	0.000250	0.215750	0.070000	
max	3.217000	2.735000	2.906000	

	Xylanimonas_cellulosilytica	Pseudomonas_trivialis	\
count	16.000000	16.000000	
mean	0.337937	0.284250	
std	0.749813	0.776747	
min	0.000000	0.000000	
25%	0.000000	0.000000	
50%	0.000000	0.000000	
75%	0.287250	0.000250	
max	2.272000	2.369000	

```
       Bacillus_cereus/thuringiensis  Bacillus_pumilus  \
count                      16.000000         16.000000
mean                        0.222188          0.093500
std                         0.574739          0.204623
min                         0.000000          0.000000
25%                         0.001750          0.000000
50%                         0.014000          0.000000
75%                         0.043250          0.025750
max                         2.138000          0.609000

       Streptococcus_sanguinis  Streptococcus_gordonii
count                16.000000               16.000000
mean                  0.053062                0.000687
std                   0.090318                0.001195
min                   0.000000                0.000000
25%                   0.000000                0.000000
50%                   0.000500                0.000000
75%                   0.054500                0.001000
max                   0.298000                0.003000

[8 rows x 37 columns]
```

[6]:

```
data = data.drop(['C_Pseudomonas_aeruginosa', 'C_Staphylococcus_schleiferi',␣
↪'C_Staphylococcus_pseudintermedius', 'C_Staphylococcus_aureus'], axis=1)
```

1.2 Pairplot to see distribution and relationships between features

[7]:

```
sns.pairplot(data, plot_kws=dict(alpha=.1, edgecolor='none'))
```

[7]:

```
<seaborn.axisgrid.PairGrid at 0x7863f95f7e50>
```

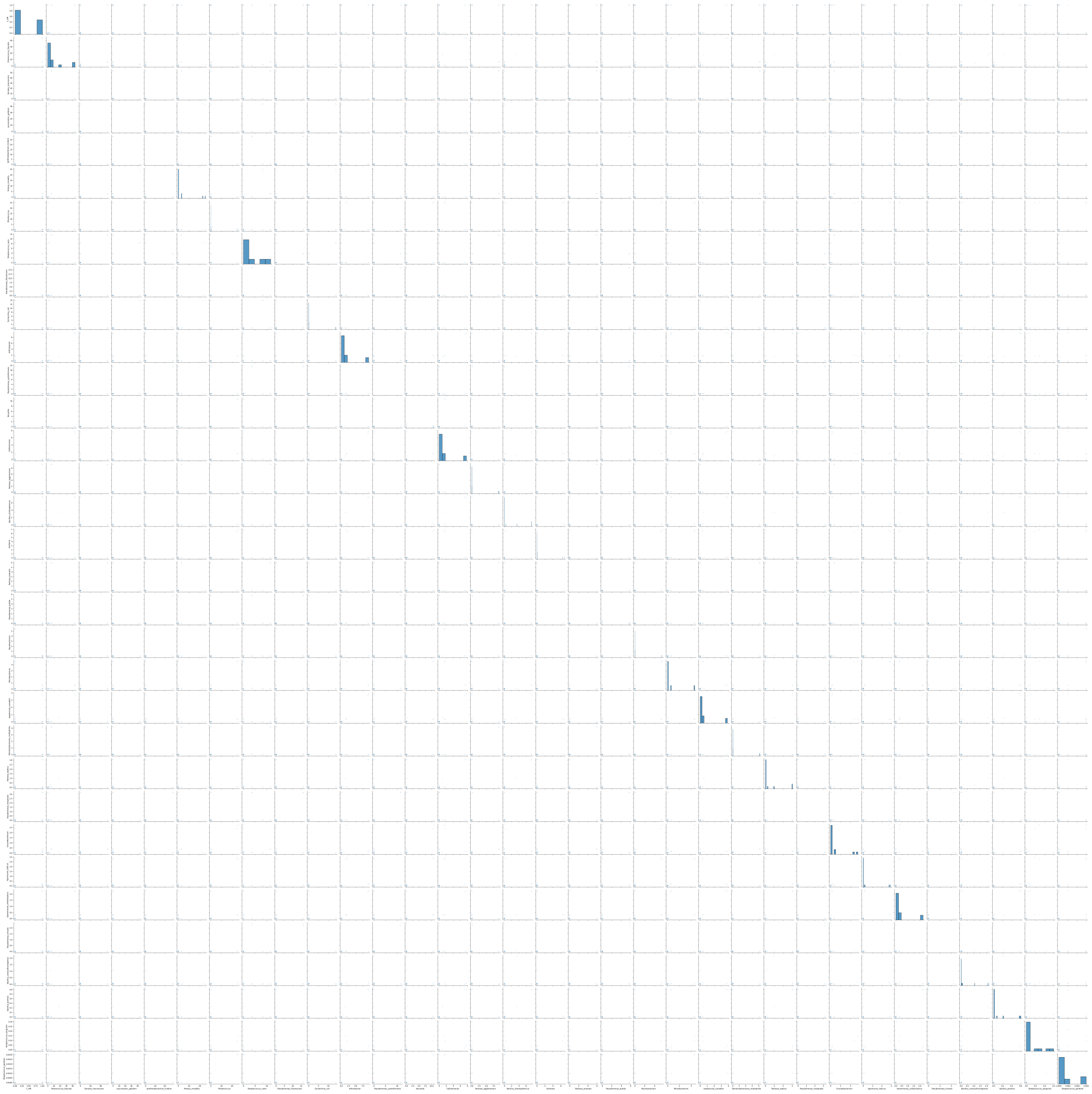

1.3 Scaling

[11]:
```
from sklearn.preprocessing import StandardScaler

y_col = "C_MR"
Y = data[y_col]
X_ss = data.drop(['name', y_col], axis=1)
#X_ss = s.fit_transform(X)
```

[12]:
```
X = pd.DataFrame(X_ss)
X.head()
```

[12]:

```
   Enterococcus_faecalis  Serratia_marcescens  Leuconostoc_gelidum  \
0                  0.010                0.000                  0.0
1                  0.062                0.000                  0.0
2                  1.708                0.000                  0.0
3                  0.150                0.488                  0.0
4                  4.576                0.000                  0.0

   Janthinobacterium_lividum  Proteus_mirabilis  Rhodococcus  \
0                        0.0              0.000        0.000
1                        0.0              0.000        0.000
2                        0.0              0.000        0.016
3                        0.0              0.018        0.000
4                        0.0              0.000        0.000

   Streptococcus_canis  Pseudomonas_fluorescens  Escherichia_coli  \
0                0.000                      0.0             0.000
1                0.000                      0.0             0.013
2                0.000                      0.0             0.046
3                0.106                      0.0             0.000
4                0.000                      0.0             0.131

   Arthrobacter  …  Pantoea_septica  Pseudomonas_marginalis  \
0         0.000  …            0.000                     0.0
1         0.002  …            0.002                     0.0
2         0.324  …            0.097                     0.0
3         0.000  …            0.006                     0.0
4         0.206  …            0.239                     0.0

   Corynebacterium  Agromyces_italicus  Xylanimonas_cellulosilytica  \
0            0.000                 0.0                        0.000
1            0.000                 0.0                        0.000
2            0.004                 0.0                        0.042
3            0.002                 0.0                        0.000
4            0.000                 0.0                        0.000

   Pseudomonas_trivialis  Bacillus_cereus/thuringiensis  Bacillus_pumilus  \
0                    0.0                          0.012             0.000
1                    0.0                          0.013             0.000
2                    0.0                          0.002             0.061
3                    0.0                          0.033             0.000
4                    0.0                          1.072             0.000

   Streptococcus_sanguinis  Streptococcus_gordonii
0                    0.038                   0.000
1                    0.000                   0.000
2                    0.298                   0.003
3                    0.214                   0.001
```

```
4                     0.000                    0.000

[5 rows x 32 columns]
```

1.4 Split in to training and test sets

[28]:
```python
from sklearn.model_selection import train_test_split

X_train, X_test, y_train, y_test = train_test_split(X, Y, test_size = 0.3)
```

helper function:

[29]:
```python
from sklearn.metrics import accuracy_score, precision_score, recall_score,␣
↪f1_score

def measure_error(y_true, y_pred, label):
    return pd.Series({'accuracy':accuracy_score(y_true, y_pred),
                      'precision': precision_score(y_true, y_pred),
                      'recall': recall_score(y_true, y_pred),
                      'f1': f1_score(y_true, y_pred)},
                      name=label)
```

1.5 Simple logistic regression

[30]:
```python
from sklearn.linear_model import LogisticRegression
LR = LogisticRegression(solver='liblinear')
LR = LR.fit(X_train, y_train)

# The error on the training and test data sets
y_train_pred = LR.predict(X_train)
y_test_pred = LR.predict(X_test)
```

[31]:
```python
from sklearn.metrics import classification_report, accuracy_score,␣
↪precision_score, recall_score
from sklearn.metrics import f1_score, roc_auc_score

cr = classification_report(y_test, y_test_pred)
print(cr)

score_df = pd.DataFrame({'accuracy': accuracy_score(y_test, y_test_pred),
                         'precision': precision_score(y_test, y_test_pred),
                         'recall': recall_score(y_test, y_test_pred),
                         'f1': f1_score(y_test, y_test_pred),
                         'auc': roc_auc_score(y_test, y_test_pred)},
                         index=pd.Index([0]))
```

```
print(score_df)
```

```
              precision    recall  f1-score   support

           0       1.00      1.00      1.00         3
           1       1.00      1.00      1.00         2

    accuracy                           1.00         5
   macro avg       1.00      1.00      1.00         5
weighted avg       1.00      1.00      1.00         5

   accuracy  precision  recall   f1  auc
0       1.0        1.0     1.0  1.0  1.0
```

1.6 Decision Tree

[32]:
```
from sklearn.tree import DecisionTreeClassifier

dt = DecisionTreeClassifier(random_state=42)
dt = dt.fit(X_train, y_train)
```

[33]:
```
dt.tree_.node_count, dt.tree_.max_depth
```

[33]:
```
(3, 1)
```

[34]:
```
# The error on the training and test data sets
y_train_pred = dt.predict(X_train)
y_test_pred = dt.predict(X_test)

train_test_full_error = pd.concat([measure_error(y_train, y_train_pred, 
 ↪'train'),
                              measure_error(y_test, y_test_pred, 'test')],
                              axis=1)

train_test_full_error
```

[34]:
```
           train      test
accuracy     1.0  0.800000
precision    1.0  0.666667
recall       1.0  1.000000
f1           1.0  0.800000
```

[35]:
```
from io import StringIO
from IPython.display import Image
from sklearn.tree import export_graphviz
import pydotplus
```

[36]:
```python
# Create an output destination for the file
dot_data = StringIO()

export_graphviz(dt, out_file=dot_data, filled=True)
graph = pydotplus.graph_from_dot_data(dot_data.getvalue())

# View the tree image
filename = 'decision_tree.png'
graph.write_png(filename)
Image(filename=filename)
```

[36]:

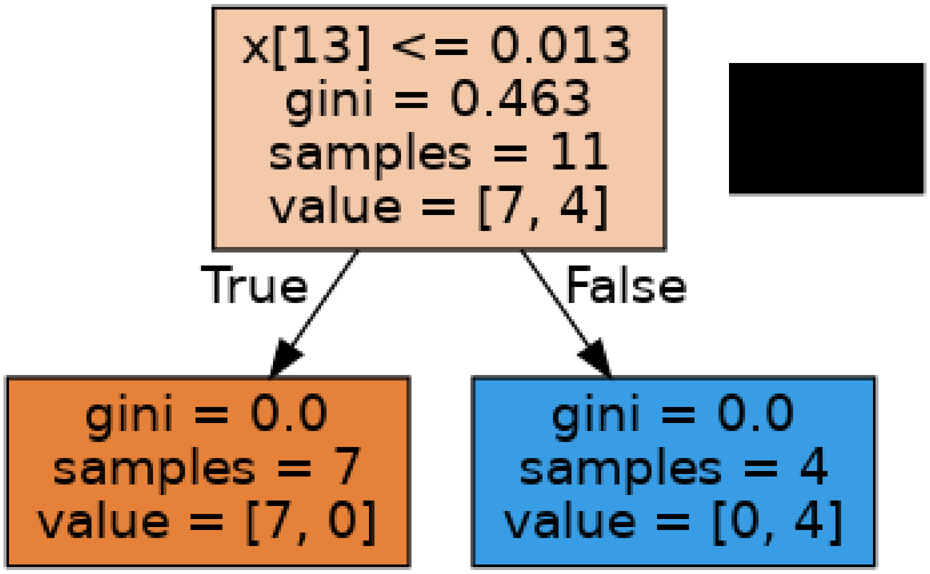

[37]:
```python
from sklearn.ensemble import RandomForestClassifier
# Suppress warnings about too few trees from the early models
import warnings
warnings.filterwarnings("ignore", category=UserWarning)
warnings.filterwarnings("ignore", category=RuntimeWarning)
```

[ ]:
```python

```

[38]:
```python
RF = RandomForestClassifier(oob_score=True,
                            random_state=42,
                            warm_start=True,
                            n_jobs=-1)

oob_list = list()

# Iterate through all of the possibilities for
# number of trees
```

```
for n_trees in [15, 20, 30, 40, 50, 100, 150, 200, 300, 400]:

    # Use this to set the number of trees
    RF.set_params(n_estimators=n_trees)

    # Fit the model
    RF.fit(X_train, y_train)

    # Get the oob error
    oob_error = 1 - RF.oob_score_

    # Store it
    oob_list.append(pd.Series({'n_trees': n_trees, 'oob': oob_error}))

rf_oob_df = pd.concat(oob_list, axis=1).T.set_index('n_trees')

rf_oob_df
```

[38]:

```
              oob
n_trees
15.0     0.090909
20.0     0.272727
30.0     0.272727
40.0     0.272727
50.0     0.272727
100.0    0.272727
150.0    0.272727
200.0    0.272727
300.0    0.272727
400.0    0.181818
```

[39]:

```
RF = RandomForestClassifier(random_state=42)
RF.set_params(n_estimators=40)

# Fit the model
RF.fit(X_train, y_train)

# The error on the training and test data sets
y_train_pred = RF.predict(X_train)
y_test_pred = RF.predict(X_test)
```

[40]:

```
cr = classification_report(y_test, y_test_pred)
print(cr)

score_df = pd.DataFrame({'accuracy': accuracy_score(y_test, y_test_pred),
                         'precision': precision_score(y_test, y_test_pred),
                         'recall': recall_score(y_test, y_test_pred),
```

```
                         'f1': f1_score(y_test, y_test_pred),
                         'auc': roc_auc_score(y_test, y_test_pred)},
                         index=pd.Index([0]))

print(score_df)
```

```
              precision    recall  f1-score   support

           0       1.00      1.00      1.00         3
           1       1.00      1.00      1.00         2

    accuracy                           1.00         5
   macro avg       1.00      1.00      1.00         5
weighted avg       1.00      1.00      1.00         5

   accuracy  precision  recall   f1  auc
0       1.0        1.0     1.0  1.0  1.0
```

[ ]:

```

```
