# Supplementary material for: Breaking the culture habit: Complementing culture-based veterinary diagnostics with metagenomic data -A case study of feline and canine skin infections
Source: BMC Vet Res. 2026 May 9;22:362. doi: 10.1186/s12917-026-05476-x (PMC13285402; doi:10.1186/s12917-026-05476-x)
Supplement: Supplementary file 5 — Supplementary Material 5. [file 12917_2026_5476_MOESM5_ESM.docx]

| Skin #  S4: AI suggested antibiotic treatments and adjustments should be regarded as research use only and not for clinical decision-making. Gene presence ≠ phenotypic resistance and requires AST and prospective validation. | VDL Finding | Initial Recommendation | MGX Data | Revised Recommendation |
| --- | --- | --- | --- | --- |
| FS1 | *Staphylococcus aureus* | Clindamycin or amoxicillin-clavulanate | BLAZ, BLAR/BLAI, FOSB, NorA/B/C, RLMH (MLS resistance) | Vancomycin or linezolid:  Avoid clindamycin (MLS resistance genes); Avoid ß-lactams (BLAZ); Avoid fosfomycin (FOSB) |
| CS1 | *P. aeruginosa + S. schleiferi* | Pseudomonas: Gentamicin/tobramycin; Staph: Clindamycin/cephalexin | OXA, PDC, MexA-O efflux systems, APH3, ANT6, extensive ß-lactamases | Pseudomonas: Colistin/polymyxin B; Staph: Vancomycin/daptomycin: Avoid aminoglycosides (APH3, ANT6); Avoid all ß-lactams (multiple ß-lactamases); Combination therapy required |
| CS2 | *P. aeruginosa* | Gentamicin or tobramycin | Similar to CS1: extensive efflux pumps, ß-lactamases, aminoglycoside resistance | Colistin or polymyxin B: Avoid aminoglycosides; Polymyxins as first-line |
| CS3 | *P. aeruginosa* | Gentamicin or tobramycin | Similar to CS1: MexA-O systems, ß-lactamases | Colistin or polymyxin B: Avoid aminoglycosides; Polymyxins as first-line |
| CS4 | *MR S. schleiferi* | Chloramphenicol or lincomycin | SRT (Class C ß-lactamase), extensive efflux systems, MLS resistance genes | Vancomycin or linezolid: More reliable systemic options; Avoid chloramphenicol (efflux) |
| CS5 | *MR S. pseudintermedius* | Chloramphenicol or lincomycin | CARB, RTG, ANT3/4, APH6, ERMB, multiple efflux pumps | Vancomycin or linezolid +/- rifampin combination: Avoid chloramphenicol; ERMB confirms MLS resistance; Consider combination therapy |
| CS6 | *S. pseudintermedius* | Cephalexin, clindamycin, or amoxicillin-clavulanate | Limited resistance genes, mainly efflux pumps, some aminoglycoside resistance | No major changes: Cephalexin, clindamycin, or amoxicillin-clavulanate |
| CS7 | *S. pseudintermedius* | Cephalexin, clindamycin, or enrofloxacin | Minimal resistance genes detected | No major changes: Cephalexin, clindamycin, or enrofloxacin |
| CS8 | *MR S. schleiferi* | Clindamycin or doxycycline | Similar to CS4: extensive resistance profile | Vancomycin or linezolid + topical mupirocin: Systemic therapy change; Add topical adjunct |
